# Roles for PI3K/AKT/PTEN Pathway in Cell Signaling of Nonalcoholic Fatty Liver Disease

**DOI:** 10.1155/2013/472432

**Published:** 2013-01-30

**Authors:** Satoru Matsuda, Mayumi Kobayashi, Yasuko Kitagishi

**Affiliations:** Department of Environmental Health Science, Nara Women's University, Kita-Uoya Nishimachi, Nara 630-8506, Japan

## Abstract

Nonalcoholic fatty liver disease (NAFLD) is the most common form of liver pathologies and is associated with obesity and the metabolic syndrome, which represents a range of fatty liver diseases associated with an increased risk of type 2 diabetes. Molecular mechanisms underlying how to make transition from simple fatty liver to nonalcoholic steatohepatitis (NASH) are not well understood. However, accumulating evidence indicates that deregulation of the phosphatidylinositol 3-kinase (PI3K)/AKT pathway in hepatocytes is a common molecular event associated with metabolic dysfunctions including obesity, metabolic syndrome, and the NAFLD. A tumor suppressor PTEN negatively regulates the PI3K/AKT pathways through its lipid phosphatase activity. Molecular studies in the NAFLD support a key role for PTEN in hepatic insulin sensitivity and the development of steatosis, steatohepatitis, and fibrosis. We review recent studies on the features of the PTEN and the PI3K/AKT pathway and discuss the protein functions in the signaling pathways involved in the NAFLD. The molecular mechanisms contributing to the diseases are the subject of considerable investigation, as a better understanding of the pathogenesis will lead to novel therapies for a condition.

## 1. Introduction

Nonalcoholic fatty liver diseases (NAFLD) represent a hepatic metabolic syndrome, which is the common broad-spectrum liver disease, and it is becoming a worldwide health problem. NAFLD ranges from nonalcoholic fatty liver to nonalcoholic steatohepatitis (NASH), which often precedes liver fibrosis, cirrhosis, and hepatocellular carcinoma. NAFLD is also associated with obesity, type 2-diabetes, and metabolic syndrome [1–5]. Insulin resistance appears to induce the fat accumulation in hepatocytes and renders the liver more susceptible to diseases [6]. In addition, reactive oxygen species (ROS), endotoxins, and inflammatory cytokines result in the disease development [7]. It is also well known that several stressors like cigarette smoke, pollutants, diabetes, hypertension, and hypercholesterolemia are all risk factors to the disease [8, 9]. The hepatic insulin resistance state of fatty liver infiltration is characterized by increased free fatty acids (FFAs), which causes lipotoxicity, impairs endothelium-dependent vasodilatation, and increases oxidative stresses. Additional metabolic risk factors include leptin, adiponectin, and plasminogen activator inhibitor-1 (PAI-1), which together lead to increased oxidative stress and endothelial dysfunction [10]. Inflammation and fibrogenesis are closely related and are major targets of the NAFLD research. To date, however, the precise molecular pathogenesis of NAFLD is still unclear.

**Figure 1:**
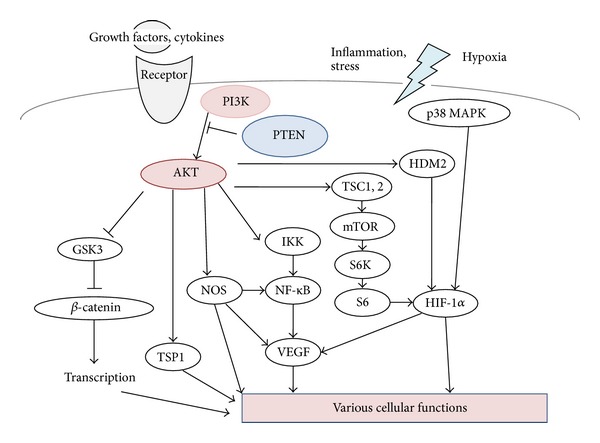
Schematic representation of PI3K/AKT/GSK3/mTOR signaling. Examples of molecules known to act on the regulatory pathways are shown. Note that some critical pathways have been omitted for clarity.

Inflammation is believed to be the chief reason behind the diseases and may cause the progression to fibrosis and subsequent cirrhosis [11, 12]. Because phosphatidylinositol-3 kinase (PI3K) and serine-threonine protein kinase AKT (also known as protein kinase B) seem to make immune cell activation by regulation of the key inflammatory cytokines [13], changes in PI3K/AKT signaling pathway may contribute to specific therapeutic effects for the NAFLD. In addition, the physiological function of PTEN is to dephosphorylate the second messengers generated by the activation of PI3K, thereby downregulating or terminating insulin signaling downstream of PI3K [14]. Therefore, potential role of the PTEN has been suggested to be involved in the development of the NAFLD. Here, we provide an overview of research on the characterization of the regulation of the PI3K/AKT/PTEN signaling (Figure 1) at the viewpoint of pathogenesis for the NAFLD. We will also interpret the current literature in an attempt to expand our understanding of the environmental and genetic causes of inflammation and its effects to the NAFLD. Intervention and therapy that alter or disrupt these mechanisms may serve to reduce the risk of the development of the disease, leading to better efficacy of new therapeutic approaches.

## 2. PI3K/AKT/PTEN Pathway Involved in Oxidative Stress and in NAFLD

Induction of ROS subjects the cells to a state of oxidative stress coupled with hepatocyte apoptosis, which is believed to play a key role in pathogenesis of NAFLD [15, 16]. In fact, hepatocyte apoptosis may be a key component of the pathogenesis involved in the progression of simple steatosis to NASH [17]. The ROS are generated during mitochondrial oxidative metabolism as well as in cellular response to inflammatory cytokines and bacterial invasion [18, 19]. Oxidative stress refers to the imbalance due to excess ROS over the capability of the cell to support effective antioxidant responses. The ROS directly interact with critical signaling molecules to initiate signaling in a variety of cellular processes, such as proliferation and survival via several signaling molecules including MAP kinases, PI3K, PTEN, and protein tyrosine phosphatases [20]. Oxidative stress then results in macromolecular damage and is implicated in various disease states such as atherosclerosis, diabetes, cancer, and aging, which is also associated with complications including NAFLD. The oxidative stress can activate a series of stress pathways involving a family of serine/threonine kinases including AKT, which in turn have a negative effect on insulin signaling [21]. In fact, experimental data suggest an inverse relationship between insulin sensitivity and the ROS levels [22, 23]. Some of the consequences of an oxidative environment are the development of insulin resistance, *β*-cell dysfunction, impaired glucose tolerance, and mitochondrial dysfunction, which can lead to the diabetic disease. Oxidative stress can be reduced by controlling hyperglycemia and calorie intake [24].

The absent expression of liver-specific PTEN may be associated with hepatic steatosis, inflammation, and fibrosis. Actually, PTEN-deficient mice have been shown to have biochemical and histological evidence of NASH [25]. The mechanism for NASH in this animal model is reported to be due to increased lipogenesis, inflammation, and fibrosis. Hypoxia accelerates the changes that have been observed in PTEN-deficient mice developing NASH [26]. The activation of AKT in PTEN-deficient cells leads to the phosphorylation of GSK3*β*, which is active in resting cells but is inactivated by the phosphorylation [27]. The GSK3*β* has been linked to the regulation of an assembly of transcription factors, including **β**-catenin, nuclear factor *κ*B (NF-**κ**B), AP-1, NF-AT, and CREB [28]. The altered activity of GSK3*β* then causes various effects on cytokine expression. Activation of PI3K then results in the inhibition of proinflammatory incidents such as expression of type I interferon, IL-12, and TNF-*α*. In addition, PI3K and mTOR seem to upregulate the anti-inflammatory cytokines [29, 30].

## 3. Function and Characterization for the PI3K/AKT/PTEN Pathway

**Figure 2:**
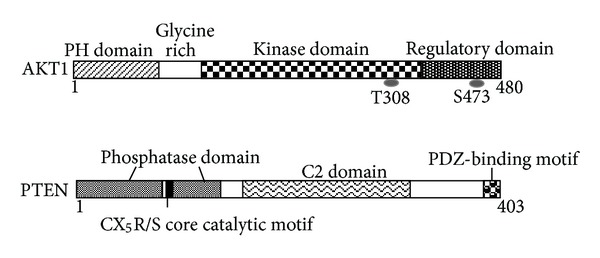
Schematic structures of AKT1 and PTEN protein. The predicted consensual domain structures for each protein are depicted. The functionally important sites including the sites of protein phosphorylation are also shown. Note that the sizes of protein are modified for clarity. PH domain: pleckstrin homology domain; C2 domain: a protein structural domain involved in targeting proteins to cell membranes; PDZ: a common structural domain in signaling proteins (PSD95, Dlg, ZO-1, etc.).

The PI3K pathways are known as regulating metabolism, cell growth, and cell survival [31]. As active form of PI3K is an oncogene, amplifications and mutations of PI3K are commonly found in many kinds of human cancers [31, 32]. The PI3K in mammalian cells forms a family that can be divided into three classes based on the structure, distribution, and mechanism of activation [33]. Class I PI3Ks are divided into class IA and class IB based on different associated adaptors. Class IA PI3Ks are activated by receptor tyrosine kinases, while class IB PI3Ks are activated by G-protein-coupled receptors. These PI3Ks are heterodimers consisting of a regulatory subunit such as p85 and a catalytic subunit such as p110. The phospholipid second messengers generated by the PI3Ks provide a common mechanism for multiple steps during intracellular signal transduction. The AKT is a major downstream target of the PI3Ks. Human AKT has three isoforms: AKT1, AKT2, and AKT3 [34]. The PIP3, a product of PI3K, binds to AKT and leads to the membrane recruitment of the AKT, and it also binds to phosphoinositide-dependent kinase 1 (PDK1) via their pleckstrin homology (PH) domains, then PDK1 phosphorylates AKT in the kinase domain (Thr 308 in AKT1). For the full activation of AKT, the phosphorylation within the carboxyl-terminal regulatory domain (Ser 473 in AKT1) of AKT by PDK2 is required [35]. Schematic structure of the predicted AKT1 protein is shown in Figure 2. Once activated, AKT moves to the cytoplasm and nucleus, where it phosphorylates, activates, or inhibits many downstream targets to regulate various cellular functions (Figure 1). AKT inhibits the GTPase-activating protein (GAP) activity of the tuberous sclerosis complex 1 (TSC1) and TSC2 complex by phosphorylating TSC2 tuberin protein, leading to the accumulation and activation of the mTOR complex (Figure 1) [36]. The mTOR mediates the phosphorylation of the ribosomal protein S6 kinases and eukaryotic translation initiation factor 4E-binding protein 1 leading to the release of the translation initiation factor eIF4E [37]. Liver-specific p70 S6 kinase depletion protects against hepatic steatosis and systemic insulin resistance [38]. The GSK3 is also a serine/threonine kinase that was initially identified as playing a role in the regulation of glycogen synthesis in response to insulin receptor stimulation. This molecule has also been shown to be involved in cellular proliferation, programmed cell death, embryogenesis, and circadian entrainment, in addition to the regulation of glycogenesis [39].

PTEN is a dual-specificity phosphatase which has protein phosphatase activity and lipid phosphatase activity that antagonizes PI3K activity [40, 41]. The human genomic *PTEN* locus consists of nine exons on chromosome 10q23.3, encoding a 5.5 kb mRNA that specifies a 403 amino acids open reading frame. The translation product is a 53 kDa protein with homology to tensin and protein tyrosine phosphatases (PTPs). Schematic structure of the predicted PTEN protein is shown in Figure 2. PTEN negatively regulates the activity of PI3K/AKT signaling through converting phosphatidylinositol 3,4,5-triphosphate (PIP3) into phosphatidylinositol 4,5-bisphosphate (PIP2). Peroxisome proliferator activated receptor **γ**, p53, and activating transcription factor 2 can transcriptionally upregulate PTEN, while transforming growth factor- (TGF-) **β**, NF-**κ**B, and Jun negatively regulate PTEN expression. Interestingly, rosemary extract represses PTEN expression in K562 leukemic culture cells [42]. PTEN activity can also be regulated by the posttranslational regulation including phosphorylation, acetylation, and oxidation [43]. PTEN protein consists of N-terminal phosphatase, C-terminal C2, and PDZ (PSD-95, DLG1, and ZO-1) binding domains. The PTEN CX5R(S/T) motif resides within an active site that surrounds the catalytic signature with three basic residues, which are critical for PTEN lipid phosphatase activity. The structure endows PTEN with its preference for acidic phospholipid substrates such as PIP3. In addition, the C-terminus of PTEN contains two PEST (proline, glutamic acid, serine, and threonine) sequences involved in protein degradation [44]. AKT activation leads to HIF-1*α* stabilization, whereas PTEN attenuates hypoxia-mediated HIF-1*α* stabilization [45]. The instability of mutant PTEN and the reduction of HIF1*α* degradation have been shown to involve protein interactions. Inhibition of the Casein kinase II-mediated PTEN phosphorylation results in increased PTEN activity and a reduction of AKT activity [46].

## 4. PI3K/AKT/PTEN Pathway Involved in Type 2 Diabetes

Type 2 diabetes is characterized by diminished pancreatic *β*-cell function. Insulin signaling within the *β*-cells has been shown to play an important role in maintaining the function of the *β*-cells. Under basal conditions, enhanced insulin-PI3K signaling via deletion of PTEN leads to increased *β*-cell mass [47]. Mice with PTEN deletion in pancreatic cells show increase the *β*-cell mass because of both increased proliferation and reduced apoptosis. In particular, the relationship between PTEN function and adipocyte-specific fatty-acid-binding protein FABP4 is of interest in *β*-cell signaling [48]. The interaction of PTEN to FABP4 suggests a role for this phosphatase in the regulation of lipid metabolism and cell differentiation [49]. In this way, tissue targeted deletion of PTEN leads to improved insulin sensitivity in the insulin-responsive tissues and protects from diabetes. In addition, PTEN has been shown to be upregulated in insulin resistance model of insulin/insulin-like growth factor-1 signaling ablation in *β*-cells [50]. PTEN expression in pancreatic islets is also upregulated in models of type 2 diabetes [51]. Furthermore, PTEN is a key negative regulator of insulin-stimulated glucose uptake in vitro and in vivo [52]. Even the partial reduction of PTEN is enough to elicit enhanced insulin sensitivity and glucose tolerance. So, PTEN exerts a critical negative effect upon *β*-cell function. Accordingly, PTEN in type 2 diabetes could be a therapeutic target to prevent the degeneration of *β*-cells.

PTEN negatively regulates the activity of PI3K/AKT signaling through converting PIP3 to PIP2. The PIP3 is the principal second messenger of the PI3K pathway that mediates receptor tyrosine kinase signaling to the survival kinase AKT. Increased levels of PIP3 at the membrane cause PH domain-containing proteins such as AKT and PDK-1 to colocalize, resulting in the kinases-mediated phosphorylation and activation [53]. The activated AKT phosphorylates target proteins involved in cell survival, cell cycling, and metabolism. Cell cycle mediators affected by the AKT and PTEN levels include the forkhead transcription factors and glycogen synthase kinase [54]. So, PTEN acts as regulator of maintaining basal levels of PIP3 below a threshold for those signaling activation. Hypoxia induces an increase in phosphorylation status of the S6 ribosomal protein. The S6 ribosomal protein belongs to the PI3K/AKT/PTEN/mTOR signaling pathway and is phosphorylated by p70 S6 kinase when this pathway is activated. It has been shown that a negative feedback loop operates from p70 S6 kinase to the upstream IRS (insulin receptor substrate)/PI3K/PDK1/AKT insulin signaling pathway, suggesting a mechanism for the development of insulin resistance [55]. The PI3K/AKT signaling may also regulate angiogenesis by several downstream targets such as NOS and GSK3**β**, which commonly upregulate HIF-1**α** expression inducing VEGF transcriptional activation. Inhibition of GSK3**β** can upregulate HIF-1**α** expression and increase **β**-catenin activity. Hypoxia induces HIF-1**α** production through the increase of its stability and induces VEGF expression in a HIF-1**α**-dependent manner [56].

## 5. PI3K/AKT Signaling Modulators Involved in the NAFLD

**Figure 3:**
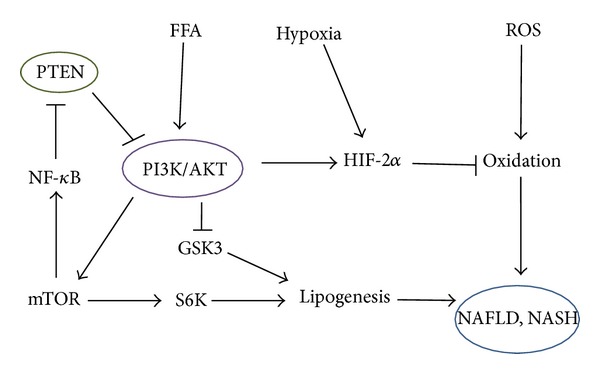
Possible potential role of free fatty acid (FFA), hypoxia, reactive oxygen species (ROS), and PTEN in the development of NAFLD and NASH based on the predominant PI3K/AKT/GSK3 pathways. Note that some critical events have been omitted for clarity.

Animals with NAFLD show histological changes including inflammation foci, increased oxidative stress, elevated serum hepatic enzyme levels, dysregulated hepatic lipid metabolism, upregulated levels of inflammatory cytokines, and apoptotic cells in the liver. Interestingly, treatment of ghrelin improves this liver injury accompanied with a restoration of PI3K/AKT pathways [57]. Ghrelin treatment alone did not influence the healthy rat liver. On the other hand, overexpression of PTEN has been shown to have inhibitory effects on insulin signaling, including decreased AKT activity and GLUT4 (glucose transporter 4) translocation to the cell membrane, showing the contribution to insulin resistance and then NAFLD progression (Figure 3) [58–60]. In contrast, downregulation of PTEN has the opposite effect with increased glucose uptake in response to insulin [61]. However, paradoxically, deletion of PTEN causes NAFLD and hepatocellular cancer [62]. The mechanism for this paradox is yet to be clarified. In PTEN-deficient mice, there is increased synthesis and storage of triglyceride in hepatocytes, due to the upregulation of PI3K/AKT activity. As a consequence of the lack of PTEN activity, there may be increased hepatocyte fatty acid uptake and increased fatty acid synthesis (Figure 3) [63].

At present, the inhibitors for PI3K/AKT signaling are as follows. Pan-PI3K inhibitors, wortmannin, and LY294002 are commonly used to inhibit cancer cell proliferation. Wortmannin is a fungal product, which exerts its effect by the covalent interaction to the conserved Lys802 of the p110**α** catalytic subunit. Both wortmannin and LY294002 cross-react with PI3K-related kinases such as mTOR and inhibit them. A p110**δ** specific inhibitor (IC486068) enhances tumor vascular destruction. The perifosine inhibits the translocation of AKT to the cell membrane. Inositol pentakisphosphate, one of the PI3K/AKT inhibitors, also inhibits tumor growth and angiogenesis. Several other AKT antagonists such as 9-methoxy-2-methylellipticinium acetate, indazole-pyridine A-443654, and isoform-specific xanthine alkaloid analogs have also been identified and shown to inhibit cancer cell growth and induce apoptosis [64]. The mTOR inhibitors such as rapamycin and its analogs inhibit mTOR activation by binding to FK506-binding protein-12 [65], which can activate upstream molecules including AKT [66]. Guanosine can afford protection against mitochondrial oxidative stress by the PI3K/AKT/GSK3 signaling and by induction of the antioxidant enzyme HO-1 [67]. Melatonin also prevents hemorrhagic shock-induced injury through an AKT-dependent HO-1 expression in animal model. Activation of PI3K/AKT/GSK3 signaling may not only upregulate the HO-1 expression, but also the protective effects of this pathway may be linked to the effects of HO-1. It is important to exploit the potential benefits of these agents for diabetes or NAFLD and/or optimal treatment and/or combination with these inhibitors.

## 6. Perspective

NAFLD is a multifactorial disease predominantly regulated by the interplay of genetic predisposition and environmental factors. It is accepted that the molecular mechanisms involved in the development and progression of the NAFLD are similar to those leading to the obesity and metabolic syndrome. In fact, the dysregulation of lipid metabolism, insulin signaling, inflammatory response, and immune response has important role in the onset and outcome of the NAFLD. The precise involvement of the PI3K/AKT//PTEN/GSK3/mTOR in signaling has remained unexplored. Between inflammation and NAFLD, there might be common pathways including the PI3K/AKT/GSK3/mTOR pathway. Whereas many questions remain to be answered about the role of the PI3K/AKT/PTEN/GSK3/mTOR signaling in the NAFLD, it is possible that inhibition of the signaling in specific hepatic cell populations could be associated with distinct behavioral outcomes. More understanding of the precise intracellular mechanisms downstream of PI3K/AKT/PTEN/GSK3/mTOR signaling changes in NAFLD could provide novel insights into the development of new therapeutic approaches having greater efficacy against NAFLD.

## Abbreviations

GAP: GTPase-activating protein
GLUT4: Glucose transporter 4
HIF-1*α*: Hypoxia-inducible factor-1*α*
HO-1: Heme oxygenase-1
IRS: Insulin receptor substrate
mTOR: Mammalian target of rapamycin
NF-*κ*B: Nuclear factor *κ*B
NAFLD: Nonalcoholic fatty liver disease
NASH: Nonalcoholic steatohepatitis
PDK1: Phosphoinositide-dependent kinase 1
PH: Pleckstrin homology
PIP2: Phosphatidylinositol 4,5-bisphosphate
PIP3: Phosphatidylinositol 3,4,5-triphosphate
PI3K: Phosphatidylinositol-3 kinase
PTEN: Phosphatase and tensin homologue deleted on chromosome 10
PTP: Protein tyrosine phosphatase
TNF-*α*: Tumor necrosis factor-alpha
TSC1: Tuberous sclerosis complex 1.
